# Supplementary material for: Elderflowers (Sambuci flos L.): A Potential Source of Health-Promoting Components
Source: Foods. 2024 Aug 16;13(16):2560. doi: 10.3390/foods13162560 (PMC11354468; doi:10.3390/foods13162560)
Supplement: Supplementary file 1 [file foods-13-02560-s001.zip › foods-3154297-supplementary.pdf]

Table S1. Retention time (Rt); MS/MS fragmentation data of major phenolics detected in elderberry flowers (*Sambuci flos*).

| | Rt (min) | [M-H]- | MS/MS (m/z) | Compound |
|---|---|---|---|---|
| 1 | 0.91 | 191.0057 | 173.0097/127.9559/111.0091 | Quinic acid |
| 2 | 1.66 | 153.0179 | | Protocatechuic acid |
| 3 | 2.04 | 137.0125 | 93.0177 | Hydroxybenzoic acid |
| 4 | 2.25 | 365.1342 | 229.4809 | Caffeoyl N-tryptophan |
| 5 | 2.77 | 341.0524 | 179.0832 | Caffeoylhexose |
| 6 | 3.35 | 353.0652 | 191.0426/179.0340 | 3-Caffeoylquinic acid |
| 7 | 4.08 | 707.1338 | 353.0586/191.0351 | Caffeoylquinic acid dimer |
| 8 | 4.27 | 353.0650 | 191.0416/179.0345 | cis-3-Caffeoylquinic acid |
| 9 | 4.59 | 353.0651 | 191.0421 | 5-Caffeoylquinic acid |
| 10 | 4.70 | 353.0649 | 173.0326 | cis-4-Caffeoylquinic acid |
| 11 | 4.75 | 577.0648 | 289.0449 | B-type procyanidin dimer |
| 12 | 4.79 | 353.0650 | 179.0216/135.0345 | 4-Caffeoylquinic acid |
| 13 | 5.03 | 595.1318 | 433.1200/301.0279 | Quercetin hexoside pentoside |
| 14 | 5.23 | 609.1134 | 447.1179/285.0545 | Kaempferol dihexoside |
| 15 | 5.40 | 337.0710 | 191.0423/163.1210 | 5-p-Coumaroylquinic acid |
| 16 | 5.72 | 625.1017 | 301.0996 | Quercetin dihexoside |
| 17 | 5.81 | 367.0796 | 191.0421/135.0239 | 3-Feruloyl-quinic acid |
| 18 | 5.94 | 367.0800 | 193.0417/173.0329/134.0259 | 4-Feruloyl-quinic acid |
| 19 | 5.99 | 609.0999 | 463.0766/301.0270 | Quercetin-3-O-rhamnosyl hexoside |
| 20 | 6.02 | 625.1566 | 463.0955/301.1147 | Quercetin dihexoside 1 |
| 21 | 6.32 | 355.1314 | 193.1740/179.0543 | Ferulic acid hexoside |
| 22 | 6.58 | 625.1138 | 463.1899/301.0098 | Quercetin dihexoside 2 |
| 23 | 6.80 | 639.1283 | 315.0308 | Isorhamnetin dihexoside |
| 24 | 6.91 | 667.0722 | 301.0153 | Quercetin acetyldihexoside |
| 25 | 7.07 | 609.1103 | 301.0194 | Quercetin-3-rutinoside |
| 26 | 7.48 | 609.1101 | 301.0141 | Quercetin-3-O-rhamnosyl hexoside |
| 27 | 7.55 | 609.1112 | 301.0143 | Quercetin-3-O-rhamnosyl hexoside |
| 28 | 7.69 | 593.1162 | 285.0682 | Kaempferol-3-rutinoside |
| 29 | 7.81 | 623.1246 | 315.2193 | Isorhamnetin-3-rutinoside |
| 30 | 7.85 | 515.0886 | 353.0653 | Dicaffeoylquinic acid 1 |
| 31 | 7.97 | 1031.1929 | 515.0885/353.0652 | Dicaffeoylquinic acid dimer |
| 32 | 8.10 | 477.0738 | 315.0272 | Isorhamnetin hexoside |
| 33 | 8.38 | 651.1488 | 609.0415/315.0275 | Isorhamnetin acetyl hexoside pentoside |
| 34 | 8.64 | 515.0874 | 353.0618 | Dicaffeoylquinic acid 2 |
| 35 | 8.71 | 515.0872 | 353.0621 | Dicaffeoylquinic acid 3 |
| 36 | 8.79 | 519.0832 | 477.0706/315.0236 | Isorhamnetin acetyl hexoside |
| 37 | 9.28 | 499.0950 | 337.0719/173.0327 | Coumaroylquinic acid derivative |
| 38 | 9.47 | 499.0953 | 353.0655/337.0733 | 3-O-caffeoyl-4-O-p-coumaroylquinic acid |
| 39 | 11.24 | 449.2379 | 285.1388 | Dihydrokaempferol-O-hexoside |
| 40 | 11.45 | 672.2575 | 271.0431 | Naringenin derivative |
| 41 | 11.57 | 433.0257 | 271.0429 | Naringenin hexoside |

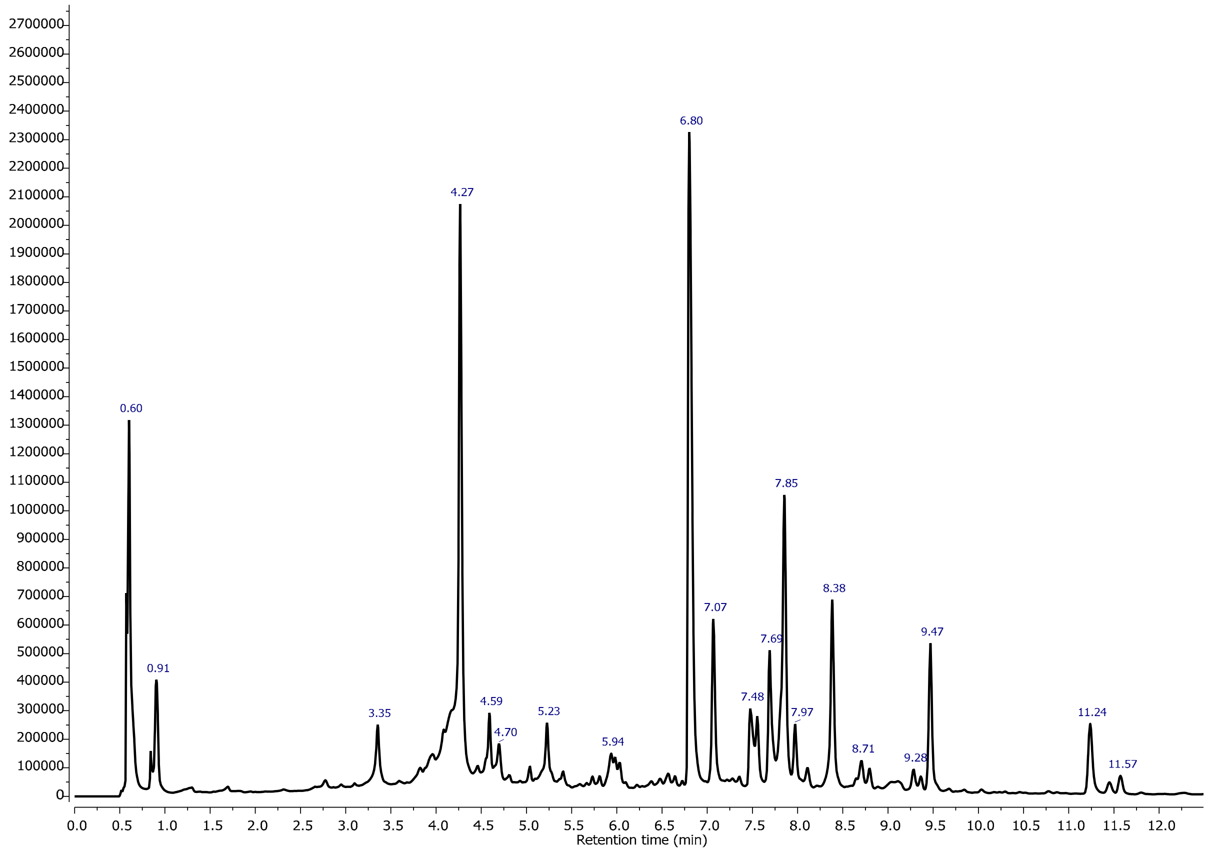


Figure S1. Chromatogram for wild elderflowers.
